# Gaps between the subjective needs of older facility residents and how care workers understand them: a pairwise cross-sectional study

**DOI:** 10.1186/s13104-016-1851-7

**Published:** 2016-01-28

**Authors:** Tomoko Ohura, Takahiro Higashi, Tatsuro Ishizaki, Takeo Nakayama

**Affiliations:** Department of Health Informatics, Kyoto University School of Public Health, Yoshida Konoe-cho, Sakyo-ku, Kyoto, 606-8501 Japan; Division of Occupational Therapy, Faculty of Care and Rehabilitation, Seijoh University, 2-172, Fukinodai, Tokai City, 476-8588 Japan; Division of Health Services Research, Center for Cancer Control and Information Services, National Cancer Center, 5-1-1, Tsukiji, Chuo-ku, Tokyo, 104-0045 Japan; Tokyo Metropolitan Institute of Gerontology, 35-2 Sakae-cho, Itabashi-ku, Tokyo, 173-0015 Japan

**Keywords:** Care for older people, Subjective needs, Older facility residents, Care workers

## Abstract

**Background:**

To promote active daily living and improve the quality of life of older facility residents, it is important that care staff understand their day-to-day activities and needs. However, only a few studies have examined the needs of older residents and how care workers understand them. This study aimed to examine the subjective needs of older residents at aged care facilities, care workers’ understanding of these needs, and the gaps that exist between them.

**Methods:**

Structured interviews with older residents with no severe cognitive impairment in ten Japanese aged care facilities and a questionnaire survey of care workers were conducted in 2008 regarding resident subjective needs. The questionnaire, which had satisfactory factorial validity, internal consistency, and reproducibility, consisted of seven items on basic activities of daily living (BADL), five items on instrumental ADL (IADL), eight items on environment and lifestyle (EL), and five items on emotion (EM). Pair-wise analyses were performed to compare responses.

**Results:**

Responses of 115 pairs were analyzed (residents ≥75 years, 85 %; 21 men, 94 women). Median proportions of residents with IADL (66 %) and EL (69 %) needs were lower compared with those with BADL (83 %) and EM (91 %) needs. Median proportions of care workers understanding IADL (55 %) and EL (60 %) needs were lower compared with those understanding BADL (87 %) and EM (87 %) needs. Less than half of the care workers understood IADL needs for household chores (30 %) and money management (43 %), and an EL need for playing a role (41 %).

**Conclusions:**

Gaps were found between resident subjective needs and how care workers understood them. Specifically, care workers underestimated older residents’ IADL and EL needs, especially with regard to playing a role. These results highlight the need for care workers to set goals based on each resident’s subjective needs and plan strategies for care provision accordingly.

**Electronic supplementary material:**

The online version of this article (doi:10.1186/s13104-016-1851-7) contains supplementary material, which is available to authorized users.

## Background

Recent years have seen a rapid increase in aging populations in developed countries [1]. Among them, Japan has the highest proportion of people aged 60 years or older in the world (32 % in 2013) [2]. Various studies, ranging from biomedical aspects to psychology and social science-related themes, have been conducted on aging [3].

The Japanese government instituted a universal long-term insurance system in 2000 [4]. The initial number of facility service users was 520,000, but this expanded to 890,000 in 2013 [5]. Various types of facilities covered by long-term care insurance for older people exist in Japan, including special nursing homes, health service facilities, and sanatorium-type medical care facilities. Special nursing homes provide regular nursing care, and sanatorium-type medical care facilities provide medical services and care. Health service facilities, which are similar to geriatric intermediate care facilities, provide rehabilitation and care, and support discharge to home.

The quality of long-term care can be evaluated from medical and technical perspectives, as well as a care receiver’s sense of satisfaction [6]. When considering care quality, comprehensive and efficient measures of care quality for older people have been developed [7], and some studies have advocated that care providers should understand the individual needs of those they care for [8, 9]. To better understand the subjective needs of aged care facility residents, a 25-item instrument for care providers to assess older people’s needs [10] was developed based on an interview study [11]. This instrument showed satisfactory factorial validity, internal consistency, and reproducibility in the context of assessing the subjective needs of institutionalized older people [10].

To improve active daily living and quality of life (QOL) of older facility residents, it is paramount that care staff understand their day-to-day activities and needs. This study aimed to examine the subjective needs of older residents at aged care facilities, care worker’s understanding of these needs, and gaps that exist between them.

## Methods

We conducted a pairwise cross-sectional study using a 25-item questionnaire [10] to measure both the subjective needs of older facility residents and care workers’ understanding of residents’ needs.

### Questionnaire

We evaluated the activities that residents wished to perform using a 25-item questionnaire [10], which was developed based on semi-structured interviews with care providers regarding care goals (2006) [11] and consideration of previous studies [12, 13]. The questionnaire encompassed the following four areas: basic activities of daily living (BADL), instrumental activities of daily living (IADL), environment and lifestyle (EL), and emotion (EM). Questions were asked in terms of “whether you want to perform each behavior regardless of the need for assistance,” rather than “whether you want assistance or not when attempting each behavior”. Residents were asked to grade each item using a five-point Likert scale (5: “strongly agree”, 4: “agree”, 3: “neutral”, 2: “disagree”, 1: “strongly disagree”) (Additional file 1). The questionnaire was validated using data collected in this study (n = 120, ${{\chi^{2} } \mathord{\left/ {\vphantom {{\chi^{2} } {\text{df}}}} \right. \kern-0pt} {\text{df}}}$ = 1.090, RMSEA = 0.03; all standardized path coefficients ranged from 0.28 to 0.87), and was determined to be reproducible using data collected in 2011 (n = 18; 14 of 25 items showed weighted kappa coefficients ≥0.60) [10]. This questionnaire [10] was also used to assess how care workers understood the needs of each resident (Additional file 2).

### Participants and study setting

**Fig. 1:**
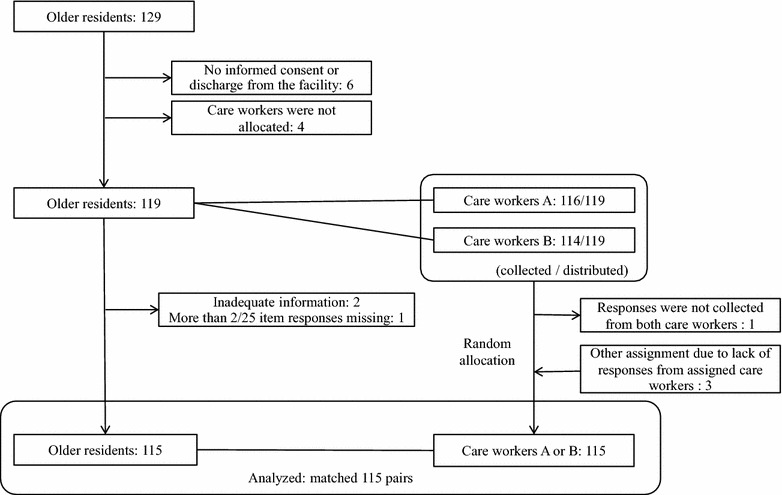
Of the 129 older residents, 119 for whom two care workers could be assigned to complete the questionnaires were included as participants of this study. After excluding four pairs, data from 115 pairs were subjected to analysis. For two pairs with no response or an insufficient response from the assigned care worker, responses from the other care worker were used

We conducted interviews with older residents of 10 facilities in Kyoto, Shiga, and Ishikawa Prefectures (two special nursing homes and eight health services facilities) (January–March 2008). A questionnaire survey was conducted with attending physicians, care managers, nurses, care workers, and rehabilitation staff (physical therapist, occupational therapist, and speech-language therapist). In this study, we only used responses from care workers who could be paired with older residents. Only older residents who were able to verbally communicate were selected arbitrarily by facility staff for this study. With respect to cognitive function, those with a Mini Mental State Examination [14] score ≤17 points (severe cognitive impairment) were excluded. Of the 129 older residents who provided consent to participate in the study, six were either discharged or withdrew their consent during the study period. Of the remaining 123 older residents, 119 for whom two care workers could be assigned to complete the questionnaires were included as participants of this study (Fig. 1). With respect to the background of the 10 facilities where the 119 older participants resided, the number of residents at each facility ranged from 84 to 240 (total, 1195 residents) at the time of the survey. The occupancy rate of each facility was between 90 and 100 %, and the number of study participants at each facility (6 to 29 residents) accounted for between 4 and 14 % of the total older residents.

### Data collection

We conducted a pilot study at two facilities before this study in order to verify the procedures. The questionnaires completed by care workers were kept in individual envelopes and collected by responsible personnel at each facility. We visited each facility to collect the questionnaires 6–8 days after distribution. We then performed individual interviews to ask each participant questions on the questionnaire form; this process reflects our effort to address potential sources of information bias for healthcare staff. If any participants were discharged from the facility, we excluded their data as well as the matched questionnaires completed by their care workers. Interviews were conducted by four healthcare professionals (one occupational therapist and three nurses) using standardized methods. Basic information including gender, age, duration of residence, Mini Mental State Examination [14], and activities of daily living (ADL) [15] was obtained from the resident records and facility staff.

Older participants were given gifts worth about 500 yen (about 4.8 USD/3.5 EUR at the time of writing), and facilities were given bookstore gift certificates according to the number of times they participated in the survey.

### Statistical analysis

We matched each older resident with two care workers who engaged in his/her care, and both care workers responded to a questionnaire regarding the resident.

We excluded pairs that provided insufficient responses to the questionnaire (i.e., two or more missing responses to the 25-item questionnaire or missing information regarding resident characteristics). If the care worker who was randomly selected out of the two assigned for each older resident did not respond to the questionnaire or had two or more missing responses, responses from the other care worker were used. Pairs for which none of the assigned care workers provided sufficient responses were excluded from the analyses.

Resident and care worker responses regarding subjective needs were evaluated on a five-point scale, and were each aggregated into two categories (4–5: with subjective needs; 1–3: without subjective needs and neutral). The proportion of residents with subjective needs and the proportion of care workers who understood the resident needs were shown along with 95 % confidence intervals (CIs) [16] for each questionnaire item.

As an additional statistical analysis, the Chi square test was performed to analyze the relationships between basic attributes and the presence or absence of resident needs, and the number of questionnaire forms completed by care workers and their basic attributes, with p < 0.05 set as a statistically significant level [17]. Furthermore, to account for multiple comparisons, the Bonferroni correction [17] was used. These analyses were performed using IBM SPSS Statistics 20 [18].

### Ethical procedure

The study protocol was approved by the Ethics Committee of Kyoto University Graduate School and Faculty of Medicine (E347). Although not all participating facilities had an ethics committee, the director of each facility approved the study, and notices of the study were posted at all facilities. The study objective was explained to the participants and/or their families, and written consent was obtained. All participating care workers were given written information, and completed questionnaires were considered their consent to participate. All collected data were subjected to linkable anonymization, and personally identifiable information, such as subject name, was never taken outside the facilities.

## Results

Data from 115 pairs were subjected to analysis. For two pairs with no response or an insufficient response from the assigned care worker, responses from the other care worker were used (Fig. 1). Resident and care worker characteristics are summarized in Tables 1 and 2. Of the 115 participants, 94 (82 %) were female, 98 (85 %) were aged 75 years or older, and 82 (71 %) were residents for 6 months and longer. The levels of independence were as follows: 77 (67 %) maintained mobility (either ambulation or wheelchair), 68 (59 %) were able to transfer to a chair or bed alone, and 56 (49 %) were able to use the lavatory (Table 1). Questionnaire forms for each of the 115 residents were filled out by 78 care workers. Of these, 49 (63 %) were female, 37 (47 %) were aged 20–29 years, and 34 (44 %) had fewer than 5 years of work experience (Table 2).

**Table 1:** Characteristics of older residents

	Resident	
	N = 115	(%)
Gender		
Female	94	(82)
Male	21	(18)
Type of facility		
NH	15	(13)
GICF	100	(87)
Age		
<75 years	17	(15)
≥75 years	98	(85)
Length of stay		
<6 months	33	(29)
≥6 months	82	(71)
Independence in ADL		
Moving: walking	44	(38)
Moving: w/c	33	(29)
Transfer	68	(59)
Using the lavatory (N = 113)	56	(49)
Eating	77	(67)
Changing clothes	55	(48)
MMSE		
≥24	60	(52)
23–18	55	(48)

*GICF* geriatric intermediate care facility, *NH* nursing home, *W/C* wheelchair, *ADL* activities of daily living, *MMSE* mini mental state examination

**Table 2:** Characteristics of care workers

	Care worker	
	N = 78	(%)
Gender		
Female	49	(63)
Male	29	(37)
Age		
20–29 years	37	(47)
30–49 years	31	(40)
≥50 years	10	(13)
Length of work		
<5 years	34	(44)
5–9 years	25	(32)
≥10 years	12	(15)
Unknown	7	(9)
Length of work at the facility		
<5 years	36	(46)
5–9 years	24	(31)
≥10 years	6	(8)
Unknown	12	(15)
Number of questionnaires for analysis		
One	52	(67)
Two	19	(24)
Three	4	(5)
Four	2	(3)
Five	1	(1)

No significant differences were found in basic characteristics (age, gender, and years of experience) of care workers by the number of older residents assessed

Table 3 shows resident responses to each questionnaire item. With regard to resident subjective needs, median proportion (minimum–maximum) was 83 % (71–94 %) for BADL needs and 91 % (87–92 %) for EM needs. In contrast, median proportion was 66 % (55–69 %) for IADL needs and 69 % (25–81 %) for EL needs. Only one item (Q19*)* in the areas of IADL and EL had a proportion higher than 80 % (81 %), and only one of the 25 items (Q15) had a proportion lower than 50 % (25 %) (Table 4).

**Table 3:** Median proportion of resident subjective needs and resident needs understood by care workers in each area

	Items	Resident subjective needs	Resident needs understood by care workers
		% (minimum–maximum)	% (minimum–maximum)
BADL	7	83 (71–94)	87 (61–97)
IADL	5	66 (55–69)	55 (30–67)
EL	8	69 (25–81)	60 (41–84)
EM	5	91 (87–92)	87 (77–92)

*BADL* basic activities of daily living, *IADL* instrumental activities of daily living, *EL* environment and lifestyle, *EM* emotion

**Table 4:** Proportion of resident subjective needs and resident needs understood by care workers

			Resident subjective needs			Resident needs understood by care workers	
			All	N1	(%, 95 % CI)	N2	(%, 95 % CI)
BADL	Q1	Go to the toilet when one wants to (includes both independently or with help)	115	108	(94, 88–97)	98	(91, 84–95)
EM	Q25	Desire to live without worry (e.g., health, food, clothing, shelter, living, and relationships)	114	105	(92, 86–96)	96	(91, 85–95)
EM	Q21	Desire to live without worrying about health	115	105	(91, 85–95)	97	(92, 86–96)
EM	Q23	Desire to live feeling good without getting depressed	115	105	(91, 85–95)	91	(87, 79–92)
EM	Q24	Desire to live enjoyable days	115	101	(88, 81–93)	88	(87, 79–92)
EM	Q22	Desire to be free of bodily pain	115	100	(87, 80–92)	77	(77, 68–84)
BADL	Q4	Desire to change clothes at one’s own pace (includes both independently and with help)	114	97	(85, 77–90)	84	(87, 78–92)
BADL	Q3	Desire to eat at one’s own pace (includes both independently and with help)	114	95	(83, 75–89)	85	(89, 82–94)
BADL	Q5	Desire to brush teeth (includes washing dentures) when one wants to (includes both independently and with help)	115	95	(83, 75–88)	78	(82, 73–89)
EL	Q19	Desire to move around for health	115	93	(81, 73–87)	53	(57, 47–67)
BADL	Q2	Take a bath when one wants to (includes both independently or with help)	115	91	(79, 71–86)	65	(71, 61–80)
BADL	Q6	Desire to move around the facility when one wants to (includes both independently and with help)	115	89	(77, 69–84)	86	(97, 91–99)
EL	Q17	Desire to carry out one’s preferred hobbies (e.g., reading, sports, games)	115	86	(75, 66–82)	55	(64, 53–73)
BADL	Q7	Desire to go outside the facility when one wants to (includes both independently and with help)	115	82	(71, 62–79)	50	(61, 50–71)
EL	Q14	Desire to talk with family or people other than staff	115	81	(70, 62–78)	68	(84, 74–90)
EL	Q20	Desire to go out to any location when one wants to (e.g., taking a walk, shopping, leisure)	115	81	(70, 62–78)	50	(62, 51–72)
IADL	Q10	Desire to interact by phone or letters when one wants to (includes both independently and with help)	115	79	(69, 60–76)	53	(67, 56–76)
EL	Q18	Desire to carry out activities that give one a role in the facility, such as manual work	115	78	(68, 59–76)	32	(41, 31–52)
IADL	Q9	Desire to go shopping when one wants to (includes both independently and with help)	115	78	(68, 59–76)	43	(55, 44–66)
IADL	Q12	Desire to cook, do laundry, and clean by oneself (includes both independently and with help)	115	76	(66, 57–74)	23	(30, 21–41)
EL	Q13	Desire to eat one’s preferred meals (includes take-out and eating out)	114	72	(63, 54–71)	56	(78, 67–86)
IADL	Q8	Desire to shave or put on makeup when one wants to (includes both independently and with help)	115	73	(63, 54–72)	44	(60, 49–71)
IADL	Q11	Desire to control money at one’s discretion	115	63	(55, 46–64)	27	(43, 31–55)
EL	Q16	Desire to talk more with staff	115	57	(50, 41–59)	30	(53, 40–65)
EL	Q15	Desire for more time to oneself and own space	114	29	(25, 18–34)	13	(45, 28–62)

Responses of residents to each item on the questionnaire are listed in descending order of percentage of residents who claimed that subjective need

*BADL* basic activities of daily living, *IADL* instrumental activities of daily living, *EL* environment and lifestyle, *EM* emotion, *N1* the number of residents who had the need, *N2* the number of care workers who understood the need (N1), *95* *% CI* 95 % confidence interval

No significant difference was found in the proportions of residents with subjective needs by gender, age, cognitive function level, level of care needed, and independence in other ADL

As shown in Table 3, care workers were less likely to understand resident needs in IADL and EL areas than in BADL and EM areas [IADL; 55 % (30–67 %), EL; 60 % (41–84 %), BADL; 87 % (61–97 %), EM; 87 % (77–92 %)]. Care workers poorly understood resident needs for IADL [Q11: keeping money at hand (43 %), Q12: performing household chores themselves (30 %)], and a need for EL [Q18: playing a role (41 %)] (Table 4).

The additional analysis revealed no significant difference in the proportions of residents with subjective needs by gender, age, cognitive function level, level of care needed, and independence in other ADL. Moreover, no significant difference was found in basic characteristics (age, gender, and years of experience) of care workers by the number of older residents assessed.

## Discussion

In this study, we measured both resident subjective needs and care workers’ understanding of resident needs, and found that while the residents had more subjective needs in the areas of BADL and EM than in the areas of IADL and EL, the care workers understood resident needs in IADL and EL areas to a lesser degree than needs in BADL and EM areas.

Most residents had common subjective needs in the areas of BADL and EM. Proportions of residents who expressed needs in the IADL and EL areas were low compared with those for BADL and EM areas. Of the 25 items, only Q15 (“Desire for more time to oneself and own space”) in the EL area had a proportion lower than 50 %. For highly independent residents, such as those in this study who were able to express their intentions, care that prioritizes resident viewpoints (e.g., care that satisfies IADL and EL needs, such as those involving fulfillment of one’s roles) will be needed in order to improve resident QOL. Previously, quality indicators focused on the older care process [19], medical management of older facility residents [20], and geriatric syndrome management [21] have been reported on resident care. In addition, six areas (home, room, social interaction, meal service, staff care, and resident involvement) have been reported as care satisfaction indicators [22]. Moreover, for older people with dementia in long-term care facilities, care providers must provide care based on the perspective of individualized care, focusing on person-centered care and understanding resident preferences [23, 24]. In recent years, an intervention study was conducted to address quality improvement in long-term care [25].

The proportions of care workers who understood resident needs in BADL and EM areas were high, compared with IADL and EL areas. Because the items in BADL and EM areas reflect basic physiological needs [26], it is possible that care workers provide support for self-care and have emotional exchanges with residents on a daily basis. One concern, however, is that care workers might provide care assuming that older residents have uniform needs, even for those who have no such needs. As suggested by our results for items in IADL and EL areas, the proportion of care workers who understood resident needs is not necessarily high in these areas. Indeed, care workers were less likely to understand subjective IADL and EL needs, which varied widely by individual or preference, than BADL and EM needs, which most residents had in common. Resident subjective needs and values are key to assessing the quality of care [21], although this might be related to the manpower of facilities and care providing systems. It is necessary to provide care based on the autonomy and dignity of older individuals with a holistic outlook [27]. Previous studies have compared perceptions of care between providers and receivers [12, 13, 28], and have revealed that the providers’ perspective differs from that of receivers. Studies in the areas of nursing and care have found that care providers tend to overemphasize needs related to aspects of their own psyche [12, 13], and there were differences in responses regarding the needs of residents, care givers, and professionals [29]. In the present study, care workers’ understanding of residents’ needs varied by area (BADL, IADL, EL and EM).

The following three needs were expressed by more than 50 % of residents, whereas less than 50 % of care workers understood them: Q11 “keep money at hand” (43 %); Q12 “perform household chores themselves” (30 %); and Q18 “need to play a role” (41 %). The former two were IADL needs and the latter, an EL need. These three items are all related to roles of residents and their demonstration of management ability, and thus linked to resident dignity. As human life activities and roles have meaning in each individual’s life, clinical practitioners including care workers should promote and enable the kind of care that allows for the maintenance of role activities based on resident values and life history. Healthcare providers should be trained to probe the psychological needs of residents in daily care [30]. To this end, care workers need to explore ways to better understand resident subjective needs, and shift mindset from care provision limited inside the facility to one that focuses on resident preferences.

This study has some limitations. First, participants were not sampled randomly but selected via convenient sampling. Although each facility staff member selected participants (potentially causing selection bias), care worker understanding of each resident’s subjective needs may have been insufficient. This underestimation of resident needs may have been even larger had we employed random sampling. Second, structured interviews were conducted by researchers, and not by usual care providers; therefore, resident subjective needs may have been excessively measured. However, this could be interpreted as having provided the opportunity for potential resident needs to rise to the surface, whereas residents might have refrained from expressing them out of consideration of the relationship with their regular care providers. Furthermore, as our participants were cognitively intact and were able to communicate verbally, application of the results to other older residents requires caution. Previous studies have reported the differences in needs of residents with dementia relative to those without dementia [31]. Finally, the present findings are based on data collected in 2008, and thus interpretation requires caution due to changes in the environment. However, as there has been almost no major policy change concerning long-term care facilities in Japan since 2008, the results of the present study are likely still valid.

## Conclusions

Most aged care facility residents had common subjective needs in the areas of BADL and EM. Proportions of residents who expressed needs in the areas of IADL and EL were somewhat low relative to those with needs in BADL and EM, although more than half of the residents had needs in these areas. This may explain why care workers were likely to understand resident needs less in IADL and EL areas than in BADL and EM areas. It will be necessary for care workers to set care goals based on an understanding of resident subjective needs, and plan policies for care provision accordingly.

## Additional files

10.1186/s13104-016-1851-7 Translated version for older residents (original questionnaire was provided in Japanese).
10.1186/s13104-016-1851-7 Translated version for staff (original questionnaire was provided in Japanese).

## Abbreviations

QOL: quality of life
BADL: basic activities of daily living
IADL: instrumental activities of daily living
EL: environment and lifestyle
EM: emotion
ADL: activities of daily living
