# Supplementary material for: Gaps between the subjective needs of older facility residents and how care workers understand them: a pairwise cross-sectional study
Source: BMC Res Notes. 2016 Jan 28;9:52. doi: 10.1186/s13104-016-1851-7 (PMC4731973; doi:10.1186/s13104-016-1851-7)
Supplement: Supplementary file 1 — 10.1186/s13104-016-1851-7 Translated version for older residents (original questionnaire was provided in Japanese). [file 13104_2016_1851_MOESM2_ESM.pdf]

Translated version for staff (Original questionnaire was provided in Japanese.)

Ⅰ. **Please choose the items that you think <u>this resident</u> may feel important <u>in current daily life</u>.**

e.g.		strongly agree	agree	neutral	disagree	strongly disagree
	Desire to be happy.	5	4 (circled)	3	2	1

	Important things in your daily life (requests for daily life assistance)	strongly agree	agree	neutral	disagree	strongly disagree
1.	Go to the toilet when one wants to (includes both independently or with help)	5	4	3	2	1
2.	Take a bath when one wants to (includes both independently or with help)	5	4	3	2	1
3.	Desire to eat at one's own pace (includes both independently and with help)	5	4	3	2	1
4.	Desire to change clothes at one's own pace (includes both independently and with help)	5	4	3	2	1
5.	Desire to brush teeth (includes washing dentures) when one wants to (includes both independently and with help)	5	4	3	2	1
6.	Desire to move around the facility when one wants to (includes both independently and with help)	5	4	3	2	1
7.	Desire to go outside the facility when one wants to (includes both independently and with help)	5	4	3	2	1
8.	Desire to shave or put on makeup when one wants to (includes both independently and with help)	5	4	3	2	1
9.	Desire to go shopping when one wants to (includes both independently and with help)	5	4	3	2	1
10.	Desire to interact by phone or letters when one wants to (includes both independently and with help)	5	4	3	2	1
11.	Desire to control money at one's discretion	5	4	3	2	1
12.	Desire to cook, do laundry, and clean by oneself (includes both independently and with help)	5	4	3	2	1
13.	Desire to eat one's preferred meals (includes take-out and eating out)	5	4	3	2	1
14.	Desire to talk with family or people other than staff	5	4	3	2	1
15.	Desire for more time to oneself and own space	5	4	3	2	1
16	Desire to talk more with staff	5	4	3	2	1
17.	Desire to carry out one's preferred hobbies (e.g., reading, sports, games)	5	4	3	2	1
18.	Desire to carry out activities that give one a role in the facility, such as manual work	5	4	3	2	1
19.	Desire to move around for health	5	4	3	2	1
20.	Desire to go out to any location when one wants to (e.g., taking a walk, shopping, leisure)	5	4	3	2	1
21.	Desire to live without worrying about health	5	4	3	2	1
22.	Desire to be free of bodily pain	5	4	3	2	1
23.	Desire to live feeling good without getting depressed	5	4	3	2	1
24.	Desire to live enjoyable days	5	4	3	2	1
25.	Desire to live without worry (e.g., health, food, clothing, shelter, living, and relationships)	5	4	3	2	1
